# Validated Smartphone-Based Apps for Ear and Hearing Assessments: A Review

**DOI:** 10.2196/rehab.6074

**Published:** 2016-12-23

**Authors:** Tess Bright, Danuk Pallawela

**Affiliations:** ^1^London School of Hygiene & Tropical MedicineLondonUnited Kingdom

**Keywords:** hearing, testing, mobile, audiometry, smartphone, applications, app, hearing loss, hearing impairment, surveys, prevalence

## Abstract

**Background:**

An estimated 360 million people have a disabling hearing impairment globally, the vast majority of whom live in low- and middle-income countries (LMICs). Early identification through screening is important to negate the negative effects of untreated hearing impairment. Substantial barriers exist in screening for hearing impairment in LMICs, such as the requirement for skilled hearing health care professionals and prohibitively expensive specialist equipment to measure hearing. These challenges may be overcome through utilization of increasingly available smartphone app technologies for ear and hearing assessments that are easy to use by unskilled professionals.

**Objective:**

Our objective was to identify and compare available apps for ear and hearing assessments and consider the incorporation of such apps into hearing screening programs

**Methods:**

In July 2015, the commercial app stores Google Play and Apple App Store were searched to identify apps for ear and hearing assessments. Thereafter, six databases (EMBASE, MEDLINE, Global Health, Web of Science, CINAHL, and mHealth Evidence) were searched to assess which of the apps identified in the commercial review had been validated against gold standard measures. A comparison was made between validated apps.

**Results:**

App store search queries returned 30 apps that could be used for ear and hearing assessments, the majority of which are for performing audiometry. The literature search identified 11 eligible validity studies that examined 6 different apps. uHear, an app for self-administered audiometry, was validated in the highest number of peer reviewed studies against gold standard pure tone audiometry (n=5). However, the accuracy of uHear varied across these studies.

**Conclusions:**

Very few of the available apps have been validated in peer-reviewed studies. Of the apps that have been validated, further independent research is required to fully understand their accuracy at detecting ear and hearing conditions.

## Introduction

In 2012, the World Health Organization (WHO) estimated that disabling hearing impairment (DHI) affects approximately 360 million people, or 5.3% of the global population [1,2]. The definition of DHI is a pure tone average (PTAv) of thresholds at 500, 1000, 2000 and 4000 hertz (Hz) in the better hearing ear of greater than 30 decibels (dB) in children, and greater than 40 dB in adults. Most people with DHI live in low- and middle-income countries (LMICs), with the greatest burden in the Asian Pacific, southern Asian, and sub-Saharan African regions [3]. The estimated global prevalence of DHI is increasing [3,4], and may be due to greater life expectancy in many countries, resulting in: increased prevalence of age-related hearing loss; early detection of hearing loss facilitated through increased availability of hearing screening equipment; increasing hearing loss due to occupational, recreational, and environmental noise exposure; and increased and extensive use of ototoxic medications for treating a range of medical conditions, such as human immunodeficiency virus (HIV) [3,4].

Hearing loss has a substantial impact on psychosocial wellbeing and economic independence [3]. If acquired in childhood, before speech has developed, hearing loss can impede language development and hence limit educational attainment [3]. Hearing loss also has high societal costs, mainly due to losses in productivity [5]. If hearing impairment is identified early and treatment is provided, many of these negative effects can be avoided [6,7]. Screening for hearing impairment can be useful for a range of age groups and patient groups, including newborns, to detect congenital hearing impairment; school children, to detect late-onset hearing impairment; the elderly, to identify age-related hearing loss (presbyacusis); and those with HIV [3,8-11]. In addition, screening for hearing impairment in population-based surveys is important to determine its magnitude and plan services accordingly [12]. However, substantial challenges exist in screening for hearing impairment (especially in LMICs) such as the need for a quiet testing environment, prohibitively expensive specialist hearing assessment equipment that requires regular calibration, and skilled professionals to conduct clinical tests. In many LMICs, there is a severe shortage of hearing health care professionals (ie, audiologists, speech pathologists, and ear, nose, and throat [ENT] specialists). In most of sub-Saharan Africa, services are either nonexistent or limited to urban centers, resulting in 1 ENT per 250,000 to 7.1 million people [13]. This scarcity contrasts with Europe, where there is 1 ENT per 10,000-30,000 people [14]. Due to these barriers, hearing impairment remains undetected and unmanaged for a substantial number of people in LMICs, and robust data from population-based surveys is lacking. 2012 WHO prevalence estimates comprised of 42 population-based surveys in 29 countries [1,2,6]. In contrast, the Rapid Assessment of Avoidable Blindness survey methodology been used in over 200 population-based surveys of visual impairment [33].

The gold standard for hearing screening for people >4 years of age is Pure Tone Audiometry (PTA) [12]. For subjects <4 years of age, objective tests such as Otoacoustic Emissions (OAE) and Auditory Brainstem Response (ABR) testing are recommended [12]. Understanding the probable causes of hearing loss is vital for management and referral processes. Causes of hearing loss are typically determined using a comprehensive battery of tests. In hearing screening programs, these tests include tympanometry (a test of middle ear function) and otoscopy (visual examination of the eardrum). The equipment and expertise required for these tests and examinations is lacking. However, new and innovative technologies that are low-cost, easy to use, and automated have recently been developed and may be useful in overcoming some of the challenges. For instance, replacing PTA (typically conducted by an audiologist) with automated computer-based audiometry can provide comparable results on threshold testing [15]. Developers of smartphone apps have begun to harness this technology to generate apps for performing self-administered hearing screening tests. In addition, apps exist for performing video otoscopy, whereby images of the eardrum are captured and may be sent to an ENT specialist to diagnose and manage ear conditions remotely. With the global rise in smartphone penetration, apps offer a promising avenue to screen for hearing impairment and assess the causes in a low-cost manner. A large number of apps for measuring ear and hearing function are thought to exist that can potentially be utilized, but their scientific validity has not been reviewed in-depth. The aim of this review is to identify available apps to screen for hearing impairment, and compare the features and peer-reviewed validation studies performed to date.

## Methods

A search was conducted to find apps for ear and hearing assessments, using the most popular commercial app stores by market share: Google Play (Android apps) and the Apple App Store (iPhone/iPad apps) [16]. Next, a review of peer-reviewed literature was conducted to determine whether any of the identified apps had been validated against gold standard measures.

### Google Play and Apple App Store Search

A search was conducted on Google Play and Apple App Store in July 2015. The main types of apps searched were those that could perform audiometry, tympanometry, OAEs, ABR testing, and otoscopy. These tests were chosen, as they can be used for assessment of ear and hearing function in a range of settings, including screening programs and population-based surveys [12]. A range of search terms were used, including clinically-recognized terms such as *audiometry* and layman’s terms such as *hearing test*. Table 1 provides a list of all search terms used.

**Table 1:** Search terms used in Google Play and Apple App Store.

Concept	Search terms used
Audiometry	audiogram
	audiology
	audiometry
	hearing exam
	hearing check
	hearing loss
	hearing problem
	hearing
	hearing test
	hear
	pure tone audiometry
Tympanometry	tympanometry
	ear
	ear nose and throat
	ENT
	ear test
	otolaryngology
	middle ear
	middle ear test
Otoacoustic Emissions	otoacoustic emissions
	OAE
	
	ABR
Otoscopy	otoscope
	otoscopy
	otorhinoendoscope
	otolaryngoscope

### Inclusion and Exclusion Criteria

App titles were initially screened for relevance to the measurement of auditory function or ear examination. Apps were excluded based on their title if it was clear that the app was not applicable. For example, in a search of *hearing test,* apps such as *Phone*, *Dog Hearing Test,* and *Motorola Gallery* were excluded based on title. Those with relevant (eg, *Hearing Test*) or ambiguous titles (eg, *iCare Health Monitor*) went through a second screening, in which they were reviewed in more detail using the descriptions in the app store and on the app’s website. Apps were included if they were self-administered or professionally administered tests of ear or hearing function. Apps were excluded if they did not focus on ear examination or audiological testing; they were not in English; they were included in the category of games, entertainment, or music; or they were intended for educational purposes.

### Literature Review of Smartphone Apps

#### Information Sources

Once the app store review was complete, a literature review was conducted in July 2015 to assess app validity testing. 6 databases were searched for peer-reviewed studies related to apps of ear and hearing function: PubMed/MEDLINE, EMBASE, Global Health, Web of Science, CINHAL, and mHealth Evidence. Comprehensive search terms for smartphone apps and auditory function were identified through MeSH and previous systematic reviews on similar topics. The names of identified apps from the commercial review were also included (see Multimedia Appendix 1). Developers of apps that were validated in peer-reviewed literature were contacted if specific information about the app was not available online.

#### Study Eligibility Criteria

Articles published between June 2007 and July 2015 were included in the search to align with the time-period during which apps have been available [17]. Any primary study identified in the app stores’ review that compared an app to gold standard methods was considered for inclusion. Studies that measured outcomes that allowed judgement of the app’s performance were included. These outcomes included: sensitivity, specificity, negative and positive predictive values, difference in pure-tone thresholds, and kappa diagnostic agreement. No restrictions were placed on study location, or types of participants included in the studies. Studies were excluded if they were not in the English language, or the study was not peer-reviewed. This review focused on the validity of apps available for download from commercial app stores. If the article did not specify the name of the app, or if the app being studied was not previously identified in the app stores’ review, the author was contacted for further information about the app and its availability. The article was included if the author could provide the app’s name and the app was available for purchase, either on Google Play, the Apple App Store, or elsewhere.

#### Study Selection

Articles were screened by two reviewers (TB and DP) first by titles, then abstract, and finally by full paper to determine eligibility.

#### Data Extraction

Data was extracted from eligible studies for the following study components:

1. *Methods*, including study design, comparison being made (ie, index test [app] and reference test [gold standard]), single or multiple smartphone devices used, headphone/transducer type, calibration methods, and test frequencies.

2. *Participants*, including age, sex, and sample size.

3. *Study location*, including country and setting.

4. *Publication details*, including year, journal, and declaration of conflicts of interest.

5. *Outcomes*, including type of outcome, definitions (eg, definition of hearing loss).

6. *Results*, including relevant measure of validity.

All data was extracted by one reviewer (TB), and checked by the second reviewer (DP) to ensure accuracy.

#### Methodological Quality of Studies

Methodological quality for each study was assessed using the Quality Assessment for Diagnostic Accuracy Studies (QUADAS-2) tool [18,19]. This tool assesses the following 4 domains:

1. *Patient selection*: assessment of study design, sampling method, and selection criteria.

2. *Index test (app)*: assessment of chosen test (app), testing method, and interpretation.

3. *Reference standard*: assessment of choice of reference standard and interpretation.

4. *Flow and timing*: assessment of time interval between index and reference tests, proportion of sample receiving reference standard, and proportion of participants included in the analysis.

Each domain was assessed in terms of risk of bias, and the first three domains were assessed in terms of concerns regarding applicability to the review question. Risk of bias and concerns regarding applicability were scored as *low*, *high*, or *unclear* using a series of signalling questions. If each signaling question had an answer of, “yes,” the domain was rated as having a low risk of bias or low concern of applicability. If any signaling question was answered, “no,” the domain was scored as high risk of bias or high concern of applicability. If any domain provided inadequate information to make a judgement, the domain was scored as, “unclear.” Each paper was reviewed independently for quality by two reviewers (TB and DP).

#### Synthesis of Results

Results from the literature review were synthesized using a narrative approach, rather than a meta-analysis, due to the heterogeneity of included studies.

## Results

### Google Play and Apple App Store Review

**Figure 1:**
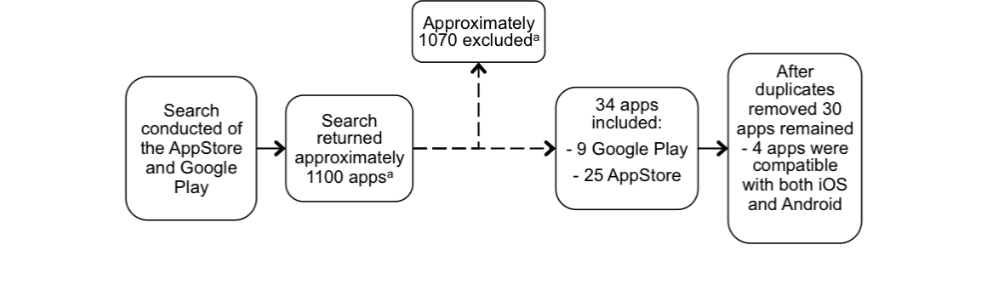
Flow diagram for apps found in app stores. Numbers are approximate due to limitations with the search platform (a=exact number of hits not provided and thus manual counting conducted).

Over 1000 apps were reviewed in the searches of Google Play and the Apple App Store, 30 of which met the inclusion criteria (Figure 1). Of these, 17% (5/30) were Android (Google) apps, 70% (21/30) were iOS (Apple) apps, and a further 13% (4/30) were compatible with both Android and iOS. Considering the function of the apps, audiometry apps formed the majority, with 26 of the 30 (87%) functioning as either self-administered automated PTA or professionally administered PTA. The remaining apps (4/30, 13%) were designed for performing otoscopy and required a separate specula phone attachment. No apps for tympanometry, OAEs, or ABR were identified. Details of the identified apps can be found in Multimedia Appendix 2.

### Literature Review of Smartphone Apps

#### Search Results

**Figure 2:**
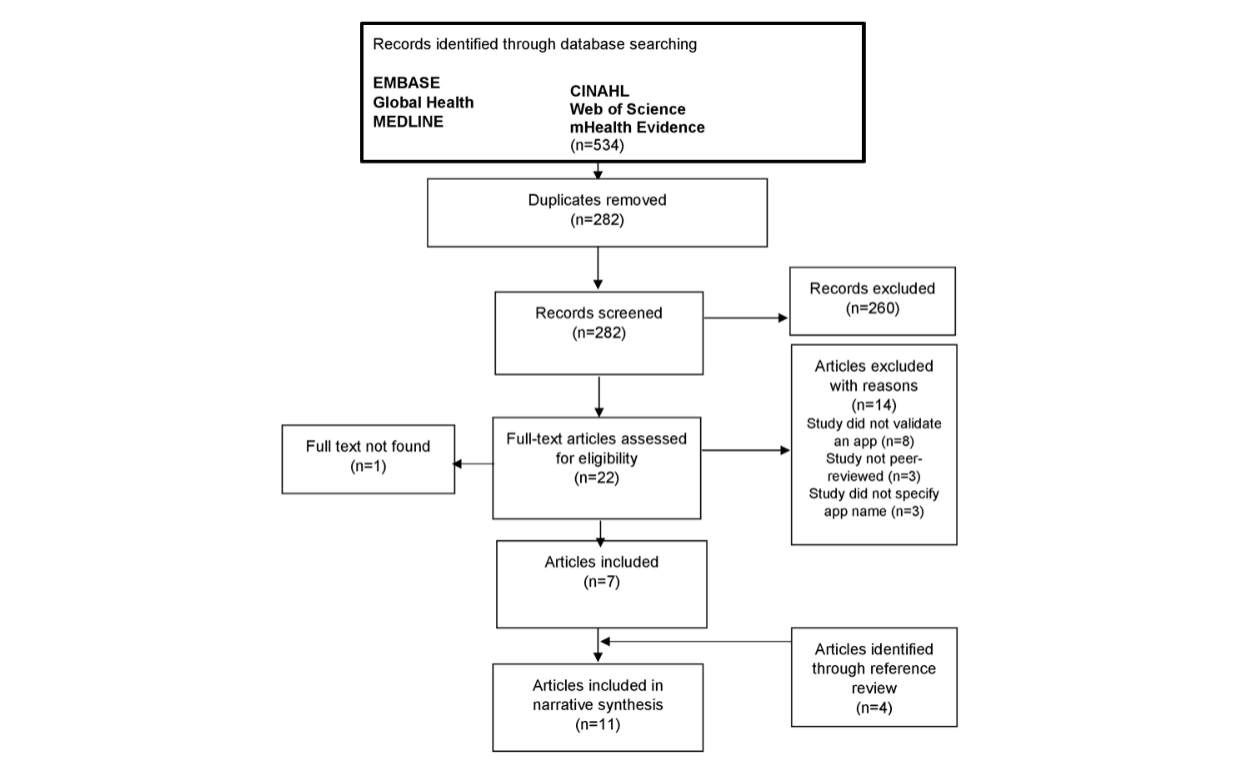
Flowchart of study selection process.

The literature review yielded 534 results: 182 in EMBASE, 157 in MEDLINE, 153 in Web of Science, 21 in CINAHL, 13 in Global Health, and 8 in mHealth Evidence. After removing duplicates across search engines, and screening titles and abstracts for relevant articles, 22 studies remained. Full text article screening resulted in 7 eligible studies. Three studies were excluded, as the app under study was not specified. Attempts were made to contact the authors of these papers for further information; however, this was not successful. Four additional studies were identified from reference lists of included articles, resulting in the inclusion of 11 studies overall (Figure 2). One further article was identified through app website review; however, the full text could not be located and therefore this article was excluded.

**Table 2:** Characteristics of apps validated in peer-reviewed literature.

App and operating system	App function	Cost (US $)^a^	Test frequency (kilohertz)	Maximum testing output (decibels)	Calibration	Transducer type and model	Additional features
uHear, iOS	Self-administered audiometry app	Free^b^	0.25, 0.5, 1, 2, 4, 6	90	Calibrated with standard Apple headphones using reference equivalent threshold sound pressure levels for TDH39 headphones (ISO389-1)	Air conduction (AC), standard apple headphones; bone conduction (BC), not measured	Noise monitoring, data storage with user identification, and questionnaire to evaluate the impact of hearing loss
shoeBOX audiometry, iOS	Self- or tester-administered audiometry app	Humanitarian $2000^c^, standard version $3100^c^, professional version $4100^c^	0.25, 0.5, 1, 2, 4, 6, 8	90-115	Calibrated with audiometric transducers using American National Standards Institute S3.6-2004 standards	AC, TDH-39 or EAR 3A insert headphones; BC, B-71 bone transducer	Noise monitoring, masking (auto calculated), and data management (cloud)
AudCal, iOS	Tester-administered audiometry app	$1.99^b^	0.5, 1, 2, 3, 4, 8	75	Calibrated for most models of iPhone/iPad using Apple headphones (standards not specified)	AC, Apple headphones; BC, not measured	Ability to export results as a photograph to photos app, and integrated with Print, Mail, and WhatsApp
hearScreen, Android	Tester-administered screening audiometry app (ie, pass/fail result)	$600^d^	1, 2, 4	40	Calibrated with nonaudiometric headphones according to ISO389-1-specified standards (within 0.1 decibel accuracy)	AC, Sennheiser HD202 headphones; BC, not measured	Noise monitoring, data capturing and sharing, and location-based referral
EarTrumpet, iOS	Self-administered audiometry app	$3.99^b^	0.25, 0.5, 0.75, 1, 1.5, 2, 3, 4, 6, 8	90-100	Calibrated with Apple’s earbuds (standards not specified)	AC, commercially available earbuds (eg, standard Apple headphones); BC, not measured	Data storage, automated masking noise, and amplification device
CellScope, iOS	Otoscopy app with separate attachment	$79^e^ for iPhone case, otoscope attachment, 4 reusable specula	Not applicable (N/A)	N/A	N/A	N/A	Port for pneumatic otoscopy

^a^Subject to change.

^b^Price excludes cost of device and transducers.

^c^Price includes transducers, software, and first year’s calibration. Price excludes the price of the iPad.

^d^Price includes device, transducers, and calibration.

^e^Price excludes cost of device.

#### Results of Included Studies

Of the 30 apps found in the review of the app stores, 5 appeared in validation studies in the peer-reviewed literature. These apps were uHear, shoeBOX audiometry, EarTrumpet, CellScope, and AudCal. One study was identified in the literature that validated an Android hearing screening app, hearScreen, that is not yet commercially available on Google Play. Thus, 6 previously validated apps were identified in the review. Of these apps, the function of 4 was self- or tester-administered PTA (uHear, shoeBOX audiometry, AudCal, and EarTrumpet), one performed screening audiometry (hearScreen; pass/fail result), and one functioned as video otoscope (CellScope).

Table 2 provides a summary of the validated apps and their specific characteristics, including function, costs, test frequencies, maximum output, calibration method, recommended transducers, and administration method.

#### Overview of Study Characteristics

The 11 selected studies are summarized in Multimedia Appendix 3 by study setting, study design, participants/sample and sample size, index (app) and reference test (gold standard), transducers and devices used, test administration method (eg, self- or tester-administered), outcome measures, calibration method, and results. Studies were performed in Canada (n=3) [20-22], Spain (n=1) [23], Israel (n=2) [24,25], USA (n=2) [26,27], and South Africa (n=3) [28-30]. The sample size of the included studies ranged from 25 to 110 participants. Participants in the included studies came from a range of age groups: adults (>18 years; n=4) [21,23,24,27], the elderly (>65 years; n=1) [25], children (<18 years; n=5) [20,26,28,30], and both children and adults (15-80 years; n=1) [29].

All included studies used a within-subjects’ study design. Ten of the 11 studies focused on comparing audiometry apps to conventional PTA [20-25,27-30], while the remaining study compared the diagnosis made with an otoscope app to traditional otoscopy [26].

Of the 10 studies validating audiometry apps, the majority carried out testing with the app in a quiet room (ambient noise levels 40-50 A-weighted decibels [dBA]; n=7) [21,24,25,27-30]. The remaining studies were performed only in a soundproof room (ambient noise <40 dBA; n=3) [20,22,23]. Three studies performed testing in multiple environments to determine the effect of ambient noise on test accuracy [21,27,29]. In terms of outcome measures, most studies (6/10, 60%) performed sensitivity and specificity analyses with defined pass/fail dB cut-offs [20-22,24,25,29]. The remaining studies (4/10, 40%) used alternative outcome measures, including the mean difference in thresholds between the app and conventional PTA [23,27,28,30]. Validation of audiometry apps in all 10 studies focused on the comparison of air conduction (AC) thresholds only, as opposed to including bone conduction (BC) threshold as well. In the single study validating the otoscopy app, Cohen’s kappa agreement was used to determine diagnostic agreement with traditional otoscopy [26].

### Summary of Main Results

#### Audiometry Apps

Of all the apps reviewed in the literature, uHear has been validated in the most studies, none of which declared a conflict of interest (n=5). Results from 3 of the 5 studies on uHear suggest that when screening for moderate or worse DHI (PTAv >40 decibels Hearing Level [dBHL]) in adults, a high sensitivity (ranging from 98.2-100%) was achieved; however, specificity was variable (ranging from 60.0-82.1%) if tests were conducted in environments with ambient noise floor at 40-50 dBA (quiet room) [21,25,29]. Ambient noise levels had significant impacts on the accuracy of uHear [21,29]. Sensitivity remained high in all test settings; however, specificity decreased in a waiting room setting (ambient noise >50 dBA) and increased when conducted in a soundproof room (ambient noise <40 dBA) [29]. Two studies concluded that uHear cannot accurately determine the precise level of hearing impairment as compared to conventional PTA, suggesting that the app could be used for screening, but not diagnostic purposes [21,25].

Two validity studies compared shoeBOX audiometry to standard pediatric audiometry, both of which declared a conflict of interest [20,22]. Sensitivity in these studies ranged from 91.2-93.3% and specificity ranged from 57.8-94.5%, depending on transducers used and test environment [20]. Individual validity studies were identified for EarTrumpet, AudCal, and hearScreen, each declaring a conflict of interest. Hearing thresholds obtained with EarTrumpet and AudCal were found to be within 10 dBHL of conventional PTA, on average [23,27]. hearScreen, a screening app that gives a pass/refer result, was found to have comparable referral rates to conventional screening audiometry [30].

#### Otoscopy Apps

Only one study focused on validating an otoscopy app. This study compared the diagnosis obtained using traditional otoscopy to that obtained using the iPhone otoscope, CellScope (n=54) [26]. This study found high levels of agreement between the two diagnostic methods. Refer to Multimedia Appendix 3 for further details of the study results.

**Table 3:** Summary of quality review of included studies (assessed using the QUADAS-2 tool) where 1 represents low risk of bias/low concern of applicability, 2 represents unclear/inadequate information to make judgement, and 3 represents high risk of bias/high concern of applicability.

Study authors (year)	Risk of bias				Applicability concerns		
	Patient Selection	Index Test	Reference Standard	Flow and Timing	Patient Selection	Index Test	Reference Standard
Abu-Ghanem et al (2015) [25]	3	1	1	1	3	1	1
Khoza-Shangase et al (2013) [28]	3	1	1	1	1	1	1
Peer et al (2015) [29]	1	1	1	3	1	1	1
Szudek et al (2012) [21]	2	1	1	1	1	1	1
Handzel et al (2013) [24]	3	3	1	1	3	3	3
Foulad et al (2013) [27]	1	1	1	1	1	1	1
Yeung et al (2013) [20]	1	1	1	3	1	1	1
Yeung et al (2015) [22]	1	3	1	3	1	1	1
Larrosa et al (2015) [23]	1	1	1	1	1	1	1
Swanepoel et al (2014) [30]	3	1	1	1	1	1	1
Richards et al (2015) [26]	3	3	1	1	1	1	1

#### Methodological Quality of Included Studies

Of the 11 peer-reviewed studies included in this review, 2 achieved a rating of *low risk of bias* and *low concern of applicability* in all domains [23,27]. The main source of bias in the included studies was selection bias. Results of the quality assessment are summarized in Table 3 and detailed in Multimedia Appendix 4.

## Discussion

Screening for hearing impairment is not feasible for many LMICs, mainly due to the dearth of skilled professionals available to conduct the required tests and high costs of specialist equipment. However, the increasing availability of apps provides an opportunity to integrate their use into screening for ear and hearing conditions in a cost effective and mobile way. This paper provides a comprehensive summary of the currently available apps for ear and hearing assessments (up to July 2015) and provides a summary of those that have been validated against gold standard measures.

Thirty commercially available apps meeting the inclusion criteria were identified on Google Play and the Apple App Store. Of these, only 5 had undergone validation, as per the peer-reviewed literature (Table 2). One additional peer-reviewed validation study referred to an Android app that is not yet available commercially. The vast majority of apps identified in the initial commercial review have not been validated against a gold standard measure in peer-reviewed literature. Most of the available apps were designed to perform audiometry (26/30, 87%) with a small proportion for otoscopy (4/30, 13%). No apps were identified for conducting OAEs, ABR, or tympanometry.

The literature review identified 11 peer-reviewed validation studies. Studies were quite heterogeneous, with variation in the cut-off level for performing sensitivity/specificity analyses, patient population, units of analysis (results of each ear separately or individual), and exclusion/inclusion criteria for participants, thus making direct comparisons across apps difficult. The quality of included studies was variable, with only 2 studies achieving a *low risk of bias* and *low concerns about applicability* in all domains (Table 3). Five peer reviewed studies were identified on uHear; however, the accuracy results varied considerably across these studies (Multimedia Appendix 3) [21,24,25,28,29]. A specificity as low as 60%, found by Abu-Ghanem et al in a quiet room setting, would result in a high rate of false positives in a screening program, and thus an unnecessary rate of referrals for diagnostic assessments, which would increase the burden on already strained health services [25]. The small sample sizes and the limited variability in degree and types of hearing loss included in the studies on uHear may limit generalizability based on the studies reviewed. Individual peer-reviewed validation studies were identified for AudCal, hearScreen, EarTrumpet, and CellScope [23,27,30]. Although the results of these studies appear to be promising, there is limited evidence to allow robust conclusions to be drawn.

Several studies demonstrated that the testing environment had a significant impact on the accuracy of results [21,27,29]. This finding is important, as ambient noise levels in screening environments are a substantial challenge and can often exceed the recommended minimum of 40 dBA [7]. Studies of audiometry apps focused on comparison with AC thresholds only, reinforcing the fact that these apps function as screening (rather than diagnostic) tools. BC testing is important for differentiating between conductive and sensorineural hearing loss; however, shoeBOX audiometry that runs on an iPad device is currently the only app compatible with BC transducers. Thus, the validity of BC testing from smartphone devices warrants further investigation. The range of frequencies that are tested in the current audiometry apps does not typically extend to 8000 Hz, thus screening for certain conditions such as ototoxicity and noise-induced hearing loss would not be possible with current app technology.

Most studies conducted tests using a single device and transducer; however, in reality there may be significant variability in results obtained with different transducer/device combinations due to issues with calibration. Annual calibration of audiometric devices is a key consideration to ensure test accuracy. Of 10 audiometry studies, only half performed calibration as part of their study [20,22,23,27,30]. This finding may be due to the fact that no standardized calibration procedure currently exists for performing tests on smartphone devices coupled with nonaudiometric headphones [30]. Several recent studies have investigated calibration methods; however, further research evidence is necessary [31,32]. Some authors suggested that poor sound attenuation provided by commercially available earbuds might have resulted in the poor accuracy of results found in nonsoundproof environments. Accuracy may improve if headphones with greater attenuation of ambient noise are utilized. However, the cost of these types of headphones can be prohibitive and calibration is still an important issue. Audiometric headphones adhering to International Organization for Standardization calibration standards (ISO389-9:2009) are vastly more expensive than commercially available headphones. Nonaudiometric supraaural headphones may assist in providing some attenuation from ambient noise. Swanepoel et al determined that Sennheiser HD202 headphones coupled to a smartphone hearing screening device can be calibrated to a professional standard using TDH-39 Reference Equivalent Threshold in Sound Pressure Levels as a reference [30]. Thus, it seems possible to use lower-cost transducers whilst ensuring test accuracy. The expertise required to professionally calibrate audiometric devices is often nonexistent in low resource settings, and equipment can remain out of calibration for lengthy periods. Hence, ongoing calibration is an additional challenge for performing accurate screening of hearing loss using apps.

Although the cost of the apps themselves are low (indeed many are free; Multimedia Appendix 2) additional costs are incurred for the device, headphones, and regular calibration. Android devices are often much less expensive than Apple products and more widely available in LMICs; however, the vast majority of available apps identified in this review were designed for Apple devices. Some of the apps identified in the literature search (shoeBOX audiometry, and hearScreen) are sold as a package including headphones, calibration for the first year, and the device (hearScreen). Although these apps appear to be higher-cost, these features allow for a level of quality control that is not currently available for apps that can be downloaded from app stores and used on various device/transducer combinations.

### Strengths and Limitations

This review has several strengths. Comprehensive search terms were identified and applied across multiple electronic databases to reduce publication bias. A clear approach to searching, screening, reviewing, and extracting data was performed independently by two reviewers. Citation bias was minimized by reviewing references of included studies. Thus, the search strategy of peer-reviewed literature is not likely to be a significant limitation.

The search of app stores was conducted using a range of search terms and the most commonly used commercial app stores were searched; however, this search had several limitations. First, unlike searches of academic databases, app store searches do not allow complex search functions such as Boolean operators or the searching of phrases such as, “hearing test.” Second, search engines did not provide the total number of hits for each search. Therefore, an estimation had to be made of the total number of apps reviewed (>1000). In addition, app store categories may not always reflect the true nature of the app, implying that some relevant apps (ie, those in the category of games) may have been missed. Furthermore, the range of search terms used may not have been fully exhaustive. For instance, alternative screening tools for hearing loss, such as self-reported questionnaires, were not included in the search. Finally, if time and resources permitted, each app would have been downloaded and tested to assess eligibility. However, this was not feasible within the scope of this study. Thus, assessment of the apps’ eligibility proved difficult in some instances if limited or vague information about the app was provided on the app stores. Given these limitations, the search of the app stores may not have been fully exhaustive, despite the range of search terms utilized and the predefined eligibility criteria.

In addition to the limitations in the app store search, given the rapid pace of app development and lengthy publication process, it might have been appropriate to broaden the search to include grey literature (eg, reports, conference papers). However, given the lack of peer review of grey literature sources, the decided methodology was justified. Finally, the review is based on an electronic search, which was completed in July 2015, and as such the review may not be entirely up-to-date.

### Future Research

This review has identified a need for further research, as many of the commercially available apps have not been validated against gold standard measures. Furthermore, many of the validated apps were not studied independently. Thus, further independent validation studies are needed for each available app for ear and hearing assessments. Studies providing a comparison of the accuracy between available audiometry apps would also be useful. The utility of telemedicine techniques, such as video otoscopy using otoscopy apps such as CellScope, could be investigated in field studies. These techniques would involve an offsite ENT, negating the need for such a specialist to be present with the patient, to help deal with the substantial human resource shortage. This additional evidence would assist in making a clear evidence-based decision about which of the apps, if any, could be recommended to be used for screening of ear and hearing conditions.

Most studies in this review focused on populations in high income countries, in which the need for validated smartphone apps still exists; however, we focused on screening for hearing impairment in low-resource settings. This discrepancy highlights the need for further research evidence for populations with DHI living in LMICs, where the greatest burden exists [2]. Finally, it is important to regularly update this review and monitor further app developments, especially for suitable apps to test pediatric populations and those who cannot perform PTA.

### Conclusions

There are a number of apps available for ear and hearing assessments; however, very few have been validated in peer-reviewed literature. Of the apps that have been validated, further independent research is required to fully understand their accuracy for detecting ear and hearing conditions. Given the results of this review, audiometry apps cannot be recommended to replace gold standard PTA conducted by an audiologist. However, despite the limited evidence obtained in this review, the portability, accessibility, self-administration, and low-cost nature of ear and hearing apps still offer an exciting opportunity to overcome the key barriers to screening for ear and hearing conditions in LMICs.

## Multimedia Appendix 1

Search strategy for EMBASE.

## Multimedia Appendix 2

Summary of apps identified on Google Play and AppStore reviews.

## Multimedia Appendix 3

Summary of selected peer-reviewed studies included in the review.

## Multimedia Appendix 4

Risk of bias of included studies.

## Abbreviations

ABR: Auditory Brainstem Response
AC: air conduction
BC: bone conduction
dB: decibel
dBA: A-weighted decibels
dBHL: decibels Hearing Level
ENT: ear, nose, and throat
HIV: human immunodeficiency virus
Hz: Hertz
LMIC: low- and middle-income country
OAE: Otoacoustic Emissions
PTA: Pure Tone Audiometry
PTAv: Pure Tone Average
QUADAS-2: Quality Assessment for Diagnostic Accuracy Studies
WHO: World Health Organization
