# Supplementary material for: Validated Smartphone-Based Apps for Ear and Hearing Assessments: A Review
Source: JMIR Rehabil Assist Technol. 2016 Dec 23;3(2):e13. doi: 10.2196/rehab.6074 (PMC5454564; doi:10.2196/rehab.6074)
Supplement: Supplementary file 1 [file rehab_v3i2e13_app1.pdf]

Appendix 1: Search strategy for EMBASE

Concept	#	Search strategy for EMBASE
**Mobile phone**	1	Mobile phone/ or MP3 player/ or smartphone*.mp. [mp=title, abstract, heading word, drug trade name, original title, device manufacturer, drug manufacturer, device trade name, keyword]
**Audiological tests**	2	exp hearing impairment/ or exp audiometry/ or exp impedance audiometry/ or exp tympanometry/ or exp pure tone audiometry/ or exp auditory screening/ or exp otoscope/ or exp otoacoustic emission/ or automated adj2 audiometry.mp. or audiogram.mp. or ABR.mp. or AABR.mp. or (automated adj1 ABR).mp. or (auditory brainstem response).mp. or (Automated auditory brainstem response).mp. or exp evoked brain stem auditory response/ [mp=title, abstract, heading word, drug trade name, original title, device manufacturer, drug manufacturer, device trade name, keyword]
**mhealth**	3	Teleaudiology.mp. telemedicine/ or mhealth.mp. [mp=title, abstract, heading word, drug trade name, original title, device manufacturer, drug manufacturer, device trade name, keyword]
**Application**	4	exp mobile application/ or app.mp. [mp=title, abstract, heading word, drug trade name, original title, device manufacturer, drug manufacturer, device trade name, keyword]
**Specific application names identified through commercial search**	5	audiotest.mp. or usound.mp or cellscope.mp. or uhear.mp or hearing test.mp. or audiometry made easy.mp. or sound check.mp. or MFA hearing test.mp or hearing game.mp. or listen carefully.mp. or TM rotator.mp. or hearing analyzer.mp. or hearing check.mp. or audiogram mobile.mp. or audiometry game.mp. or hearing exam.mp. or hearing test #1.mp. or hearing test pro.mp. or eartone.mp. or u hearing test.mp. or mimi hearing test.mp. or audCAL.mp. or iaudiometry.mp. or iaudiometer.mp. or shoeBOX audiometry.mp. or ear werx.mp or otoscope app.mp. or audicus.mp. or audiometer for android.mp. [mp=title, abstract, heading word, drug trade name, original title, device manufacturer, drug manufacturer, device trade name, keyword]
	6	1 AND 2 AND 4
	7	1 AND 2 AND 5
	8	2 AND 3
	9	1 AND 2
	10	limit 6/7/8/9/10 to (human and english language and yr="2007 -Current")
