# Supplementary material for: Validated Smartphone-Based Apps for Ear and Hearing Assessments: A Review
Source: JMIR Rehabil Assist Technol. 2016 Dec 23;3(2):e13. doi: 10.2196/rehab.6074 (PMC5454564; doi:10.2196/rehab.6074)
Supplement: Supplementary file 2 [file rehab_v3i2e13_app2.pdf]

Appendix 2: Summary of apps identified on Google Play and AppStore reviews.

Name (Developer)	Intended age group	Intended tester	Operating system	Recommended transducer	Cost ($US)[c]	Validated in peer-reviewed literature
**Audiometry apps**						
Hearing test (Audiology-online)	Adults	Self-administered	Android	None specified	Free	N
Audiometry made easy (Reece Stevens)	Adults	Self-administered	Android	None specified	Free	N
Hearing Game (Mobile medico)	Children	Administered by parent/carer	Android	Phone speakers, insert headphone	$2.60	N
Audio-test (leo4google)	Adults	Self-administered	Android	None specified	Free	N
Audiometer for Android (Sandroid studio)	Adults	Self-administered or professional	Android	None specified	Free	N
Hearing Analyzer - for Audiophile, Hearing Analyzer for Audiophile Lite (TOON)	Adults	Self-administered	iOS	Apple earbuds	$2.99	N
uHear (Unitron Hearing)	Adults	Self-administered	iOS	None specified	Free	Y
Ear Werx (Brad May)	Adults	Self-administered	iOS	Apple earbuds with microphone	Free/ $0.99	N
Ear Werx Premium edition (Brad May)	Adults	Self-administered	iOS	Apple earbuds with microphone	$0.99	N
Audicus hearing test lite (Audicus)	Adults	Self-administered	iOS	None specified	Free	N
Audiogram Mobile (Vincenzo Cocciolo)	Adults	Professional	iOS	Any good quality headphones, app has inbuilt calibration function	$28.99	N
Audiometry game (Wevosys Franke)	Children	Self-administered	iOS	Apple earbuds	Free	N
Hearing Check (Bxtel)	Adults	Self-administered	iOS	None specified	Free	N
Hearing exam (Hyunmin Park)	Adults	Self-administered	iOS	None specified	Free	N
Hearing test #1 (Aveos)	Adults	Self-administered	iOS	None specified	$1.80	N

Name<br>(Developer)	Intended age group	Intended tester	Operating system	Recommended transducer	Cost ($US)[c]	Validated in peer-reviewed literature
**Audiometry apps**						
Hearing test pro free<br>(Up with apps)	Adults	Self-administered	iOS	None specified	Free	N
ShoeBOX Audiometry, ShoeBox Audiometry Pro<br>(Clearwater Clinical)	All ages	Professional	iOS	TDH-39, EAR 3A insert headphones, B-71 bone transducer	Humanitarian price: $2000<br><br>Standard version price: $3100<br><br>Professional version price: $4100<br><br>(includes: headphones, software, first year’s calibration. Excludes the price of the iPad)	Y
iAudiometer Lite, iAudiometer CS Lite, iAudiometer A, iAudiometer PRO, iAudiometer BAC, iAudiometer CS, iAudiometer S, iAudiometer TEP, iAudiometer PED, iAudiometer B, iAudiometer Psst!<br>(MelMedtronics)	All ages	Professional	iOS	TDH-39, TDH-49, DD45, ER-3, ER-5 and B71	Varied	N
AudCAL, AudCAL HD, AudCAL mini<br>(Jorge Alberto Rey Martinez)	Adults	Professional	iOS	None specified	Free	Y
Mimi hearing test<br>(Mimi hearing technologies)	Adults	Self-administered	iOS	Apple earbuds/pods	Free	N
uHearingTest<br>(University of California)	Adults	Self-administered	iOS	iPhone 5 Headphones	Free	N
Eartone<br>Manut (Utoomprurkporn)	Adults	Self-administered	iOS	None specified	Free	N
EarTrumpet<br>(Praxis Biosciences)	Adults	Self-administered	Android and iOS	Apple earphones with microphone	$3.99	Y

Name (Developer)	Intended age group	Intended tester	Operating system	Recommended transducer	Cost ($US)[c]	Validated in peer-reviewed literature
**Audiometry apps**						
Sound check, Listen Carefully (Starkey Hearing Technologies)	Adults	Self-administered	Android and iOS	None specified	Free	N
MFA hearing test (Unlimiter Inc)	Adults	Self-administered	Android<br>iOS	None specified	Free	N
u sound (newbrick SA)	Adults	Self-administered	Android<br>iOS	None specified	1 year $30, 5 years $105, 3 years $75	N
**Otoscopy apps**						
CellScope (CellScope)	All ages	Professional	iOS	N/A	Free<br>$79 for iPhone attachment	Y
TM-Rotator (Rockodile)	All ages	Professional	iOS	N/A	Free	N
ClearSCOPE (Clearwater Clinical Limited)	All ages	Professional	iOS	N/A	Free	N
OtoscopeApp (Austin Rose)	All ages	Professional	iOS	N/A	Free	N

[c]Subject to change
