# Supplementary material for: Validated Smartphone-Based Apps for Ear and Hearing Assessments: A Review
Source: JMIR Rehabil Assist Technol. 2016 Dec 23;3(2):e13. doi: 10.2196/rehab.6074 (PMC5454564; doi:10.2196/rehab.6074)
Supplement: Supplementary file 3 [file rehab_v3i2e13_app3.pdf]

Study author, year	Setting	Study design	Participants, Sampling method	Device(s) and transducers used, administration method	Reference/ gold standard	Outcomes	Definition of hearing loss used for sensitivity/specificity analysis	Test frequencies (kHz)	Calibration performed	Result summary
**Studies validating uHear**										
Abu-Ghanem et al. (2015)	Retirement facility.<br><br>Israel, urban	Within subjects	N=26<br>Age: 65-94 years; mean 84.4 years<br>Gender: 31% (M) 69% (F)<br>Sampling: Voluntary participation.	Single iPhone 4S<br><br>Sennheiser CX300 headphones in three available sizes with replaceable rubber covers<br><br>Self-administered in quiet room	Conventional PTA on portable audiometer in quiet room (ambient noise 41-42dBA) with audiologist	Sensitivity/ Specificity; Mean difference in thresholds; Pearson's correlation coefficient (for validity of uHear questionnaire); Test duration	PTAv>40dB HL at 500, 1000, 2000 and 4000Hz in better ear	0.25, 5, 1, 2, 4, 6	No<br><br>Single device and headphone combination was used to test all participants in order to avoid potential inter-device variability	Sensitivity=100%<br>Specificity=60%<br><br>Mean difference in thresholds varied from 0.38-17.02dB HL greater for uHear depending on the frequency. Difference statistically significant at all frequencies except 2000Hz.
Khoza-Shangaze et al. (2013)	School. South Africa, urban	Within subjects	N=86<br>Age: 8-10 years; mean 9.0 years<br>Gender: 49% (M) 51% (F)<br>Sampling: Quota	Single iPod touch<br><br>Standard earbud headphones<br><br>Administered by audiologist in a quiet room	Conventional PTA in quiet room with audiologist	Mean difference in thresholds – greater than 10dB was considered a significant shift in thresholds	Hearing level >15dB HL	0.25, 5, 1, 2, 4, 6, 8	No<br>Single device and headphone combination was used to test all participants in order to avoid potential inter-device variability	Mean difference in thresholds: Varied from 9.2 to 23.4dB HL greater for uHear depending on the frequency (standard deviation 9.4-21.9dB). Differences statistically significant at all frequencies.

Appendix 3: Summary of selected peer-reviewed studies included in the review

Study author, year	Setting	Study design	Participants, Sampling method	Device(s) and transducers used, administration method	Reference/ gold standard	Outcomes	Definition of hearing loss used for sensitivity/specificity analysis	Test frequencies (kHz)	Calibration performed	Result summary
**Studies validating uHear**										
Peer et al. (2015)	ENT clinic<br><br>South Africa, urban	Within subjects	N=25 patients<br>Age: 15-80 years; mean 43 years<br>Gender: 52% (M) 48% (F)<br>Sampling: Consecutive	Single iPhone 4<br><br>Standard earbud headphones<br><br>Self-administered in 3 different settings: quiet room, waiting room, soundproof room. Investigator was present to ensure test was completed.	Conventional PTA in soundproof room with an audiologist	Sensitivity/Specificity; Cohen's kappa comparing agreement between thresholds at different frequencies	PTAv>40dB HL at 500, 1000, 2000 and 4000Hz in either ear	0.25, 5, 1, 2, 4, 6	No<br><br>Single device and headphone combination was used to test all participants in order to avoid potential inter-device variability	**Waiting room:**<br>Sensitivity=100%<br>Specificity=64%<br>Cohen's kappa:<br>*Low frequencies:* poor to moderate<br>*High frequencies:* moderate<br><br>**Quiet room:**<br>Sensitivity=100%<br>Specificity=74%<br>Cohen's kappa:<br>*Low frequencies:* poor to moderate<br>*High frequencies:* Moderate to good<br><br>**Soundproof room:**<br>Sensitivity=100%<br>Specificity=88%<br>Cohen's kappa<br>*Low frequencies:* Poor to good<br>*High frequencies:* Moderate to very good

Study author, year	Setting	Study design	Participants, Sampling method	Device(s) and transducers used, administration method	Reference/ gold standard	Outcomes	Definition of hearing loss used for sensitivity/specificity analysis	Test frequencies (kHz)	Calibration performed	Result summary
**Studies validating uHear**										
Szudek et al. (2012)	Single otology practice within hospital.<br><br>Canada, urban	Within subjects	N=100<br>Age: 20-91 years; mean 46 years<br>Gender: 33% (M) 67% (F)<br>Sampling: Not reported	Single iPod touch<br><br>Standard earbud headphones<br><br>Self-administered in soundproof room and quiet room (ambient noise <50dBA) under supervision of clinician/researcher	Conventional PTA in a soundproof room with a single audiologist	Sensitivity/ Specificity; Mean difference in thresholds – within 10dB considered normal variation; Test duration	PTAv>40dB HL at 500, 1000, 2000 and 4000Hz<br>Ear was unit of analysis	0.25, 5, 1, 2, 4, 6	No<br>Single device and headphone combination was used to test all participants in order to avoid potential inter-device variability	**Quiet room:**<br>Sensitivity=98% (95%CI=89-100)<br>Specificity=82% (95%CI=75-88)<br><br>Mean difference in thresholds: 14dB HL greater with uHear for all ears compared to PTA, 6dB HL for ears with a hearing loss (p<0.001)<br><br>**Soundproof room:**<br>Sensitivity=100% (95%CI=92-100)<br>Specificity=90% (95%CI=83-94)<br><br>Mean difference in thresholds: 8dB greater with uHear for all ears compared to PTA, 4dB for ears with a hearing loss (p<0.001)

Study author, year	Setting	Study design	Participants, Sampling method	Device(s) and transducers used, administration method	Reference/ gold standard	Outcomes	Definition of hearing loss used for sensitivity/specificity analysis	Test frequencies (kHz)	Calibration performed	Result summary
**Studies validating uHear**										
Handzel et al. (2013)	Emergency department<br><br>Israel, urban	Within subjects	N=32<br>Age: 20-82 years; mean 51.4 years<br>Gender: 75% (M) 25% (F)<br>Sampling: Consecutive	Single iPod touch<br><br>Creative EP 630 earbud speakers with replaceable rubber covers.<br><br>Self-administered in a quiet room (ambient noise varied between 41-42dBA) supervised by researcher	Conventional PTA in soundproof room	Sensitivity/Specificity	Sudden sensorineural hearing loss: hearing loss of 2 or more hearing grades in 3 consecutive test frequencies.	0.25, 5, 1, 2, 4, 6	No<br><br>Single device and headphone combination was used to test all participants in order to avoid potential inter-device variability	Sensitivity=76% (53-92)<br>Specificity=91% (59-99)
**Studies validating Ear Trumpet**										
Foulad et al. (2013)[a]	Medical<br><br>USA, urban	Within subjects	N=42<br>Age: 20-85 years; mean 58 years<br>Gender: 55% (M) 45% (F)<br>Sampling: Consecutive	Either an iPhone or iPod (multiple devices used)<br><br>Standard earbud headphones<br><br>Self-administered in soundproof room and quiet room	Conventional pure tone audiometry in soundproof room	Percent of thresholds falling within 0-5dB, 0-10dB and 0-20dB of standard audiometry; Paired threshold differences; iOS device compatibility (how the output varies across devices)	None given	0.25, 0.5, 0.75, 1, 1.5, 2, 3, 4, 6, 8	iOS based hearing test was designed and calibrated with Apple's earbuds connected to a soundmeter through 1" tube simulating ear canal	Quiet room: Percent of thresholds falling with 10dB of standard audiometry =94% (87-100)<br><br>Soundproof room: Percent of thresholds falling with 10dB of standard audiometry =96% (91-100)

Study author, year	Setting	Study design	Participants, Sampling method	Device(s) and transducers used, administration method	Reference/ gold standard	Outcomes	Definition of hearing loss used for sensitivity/specificity analysis	Test frequencies (kHz)	Calibration performed	Result summary
**Studies validating shoeBOX audiometry**										
Yeung et al. (2013)[a]	Audiology clinic.<br><br>Canada, urban	Within subjects	N=70 children<br>Age: 3-13 years; mean 5.2 years<br>Gender: Not described<br>Sampling: Consecutive	Single iPad<br><br>Calibrated TDH-39 headphones<br><br>Soundproof room with two audiologists	Traditional play audiometry in soundproof room	Sensitivity/ Specificity; Repeated measures analysis (model); Participant performance – including time to completion, feedback from the audiologists	Threshold greater than 25dB HL in any of 4 test frequencies (500, 1000, 2000 and 4000Hz)	0.5, 1, 2, 4	Professionally calibrated by Genie Audio Inc to ANSI S3.6-2004 standards.	Sensitivity =93.3% (95%CI=71.7-99.6)<br>Specificity =94.5% (95%CI=88.6-96.3)
Yeung et al. (2015)[a]	Audiology clinic.<br><br>Canada, urban	Within subjects	N=79<br>Age: 5-17 years; mean 9.5 years<br>Gender: Not described<br>Sampling: Consecutive	Single iPad 2<br><br>Apple In Ear Headphones or Apple Earbud Headphones<br><br>Self-administered by child in quiet room supervised by an adult and a communication disorders assistant	Traditional play audiometry in sound proof room	Sensitivity/ Specificity; Receiver Operating Curve (ROC) curve to determine optimum discrimination threshold	Threshold greater than 25dB HL in any of 4 test frequencies, but changed to 30dB HL in analysis stage.	0.5, 1, 2, 4	Professionally calibrated by Genie Audio Inc to ANSI S3.6-2004 standards using non-standard RETSPLs derived using the TDH39 headphones as a reference (no standard exists for ear-bud headphones in ANSI	Sensitivity =91.2% (95% CI=78.5-97.6)<br>Specificity =57.8% (95%CI=48.2-62.6)

									S3.6-2010 document).	

Study author, year	Setting	Study design	Participants, Sampling method	Device(s) and transducers used, administration method	Reference/ gold standard	Outcomes	Definition of hearing loss used for sensitivity/specificity analysis	Test frequencies (Hertz)	Calibration performed	Result summary
**Studies validating AudCal**										
Larrosa et al. (2015)[a]	9 ENT clinics.<br><br>Spain, urban	Within subjects	N=110<br>Age: 18-91 years; mean 43.9 years<br>Gender: 54% (M) 46% (F)<br>Sampling: Consecutive	Various apple devices (iPhone 4, 5, 5c, 5s, iPad 2)<br><br>Standard earbud headphones<br><br>Audiologist administered in "minimal ambient noise level sound conditions"	Conventional pure tone audiometry in soundproof room	Cohen's kappa coefficient (agreement between both methods); Threshold compared using: Cronbach's alpha (reliability of application), Intraclass correlation coefficient (concordance), mean difference in thresholds	PTA>20dB HL at 500, 1000, 2000, 4000Hz	0.5, 1, 2, 3, 4, 8	Calibrated by researchers using iPhone 5 and EarPod (human sensitivity model – manual comparison between audiometer and standard headphones and EarPod headphones coupled with iPhone)	Kappa coefficient = 0.894<br><br>Cronbach's alpha: All cases 0.96 (≥0.80 considered excellent)<br><br>Intraclass correlation coefficient (concordance) All cases 0.93 (≥0.90 considered excellent)<br><br>Mean difference in thresholds: 0.21±6.38dB
**Studies validating hearScreen**										
Swanepoel et al. (2014)[a]	School.<br><br>South Africa, urban	Within subjects	N=162<br>Age: 5.6 – 7.7 years; mean 6.5 years<br>Gender: Not described<br>Sampling: Convenience	Two Samsung Galaxy pocket plus S5301 smartphones<br><br>Sennheiser HD202 supra-aural headphones<br><br>Administered by audiology student in quiet	Conventional screening audiometry	Agreement between smartphone and conventional screening; Referral rate; Validity of smartphone calibration; Validity of environmental noise	Threshold >25dB HL at 1000, 2000, or 4000Hz	1, 2, 4	Calibrated professionally coupled to Sennheiser HD202 headphones. Calibration within +/- 3dB of RESPLs	Smartphone and conventional hearing screening methods were in agreement in 97.8% of cases. hearScreen had a referral rate of 4.3% whilst conventional screening 3.7%.

				room. Supervised by audiologist.		monitoring; Test duration				

Study author, year	Setting	Study design	Participants, Sampling method	Device(s) and transducers used, administration method	Reference/ gold standard	Outcomes	Definition of hearing loss used for sensitivity/specificity analysis	Test frequencies (Hertz)	Calibration performed	Result summary
**Studies validating CellScope**										
Richards et al. (2015)	Academic paediatric emergency department<br><br>USA, urban	Within subjects	N=51<br>Age: mean 5.1 years<br>Gender: 53% (M) 47% (F)<br>Sampling: Convenience.	**CellScope** otoscopy	Traditional otoscopy with camera attachment	Ear examination findings were grouped as normal, abnormal with effusion, abnormal with erythema of TM, abnormal (other not listed), cerumen impaction, and unable to visualise. Inter and intrarater diagnostic agreement.	N/A	N/A	N/A	*Intrarater agreement resident physicians:* Right ear: k=0.74 (95% CI, 0.58-0.89)<br>Left ear: k=0.74 (95% CI, 0.58-0.89)<br>*Intrarater agreement attending physicians:*<br>Right ear: k=0.86 (95% CI, 0.72-0.98)<br>Left ear: k=0.79 (95%CI,0.65-0.94)

[a]=conflict of interest declared
PTAv=Pure Tone Average
ENT= Ear Nose and Throat
RETSPLs=Reference Equivalent Threshold Sound Pressure Level
ANSI= American National Standards Institute
CI=Confidence Interval
k=Kappa
Hz=Hertz
dB=Decibels
