# Supplementary material for: Validated Smartphone-Based Apps for Ear and Hearing Assessments: A Review
Source: JMIR Rehabil Assist Technol. 2016 Dec 23;3(2):e13. doi: 10.2196/rehab.6074 (PMC5454564; doi:10.2196/rehab.6074)
Supplement: Supplementary file 4 [file rehab_v3i2e13_app4.pdf]

Study author (Year)	Risk of bias				Applicability Concerns		
	Patient Selection	Index Test	Reference Standard	Flow And Timing	Patient Selection	Index Test	Reference Standard
Abu-Ghanem (2015)[22]	High: voluntary participation	Low	Low	Low	High: elderly participants only	Low	Low
Khoza-Shangase (2013)[25]	High: quota sampling	Low	Low	Low	Low	Low	Low
Peer (2015)[26]	Low	Low	Low	High: excluded some results from analysis. Conventional PTA performed within a two-week period prior to index test. Risk that hearing levels may have significantly changed when index test was performed.	Low	Low	Low
Sduzek (2012)[19]	Unclear: details of method of selection not reported	Low	Low	Low	Low	Low	Low

Handzel (2013)[21]	Low	Low	Low	Low	High: patients with unilateral sudden sensorineural hearing loss	High: definition of hearing loss	High: definition of hearing loss
Foulad (2013)[24]	Low	Low	Low	Low	Low	Low	Low
Yeung (2013)[17]	Low	Low	Low	High: excluded results from analysis (18%)	Low	Low	Low
Yeung (2015)[18]	Low	High: pre specified threshold but adjusted in analysis	Low	High: results at 500Hz excluded from analysis	Low	Low	Low
Larrosa (2015)[20]	Low	Low	Low	Low	Low	Low	Low

Swanepoel (2014) [27]	High: convenience sampling	Low	Low	Low	Low	Low	Low
Richards (2015)[23]	High: recruitment on specific days, convenience sampling	High: reference standard performed prior to index and not blinded.	Low	Low	Low	Low	Low
High = high risk of bias/high concern of applicability, Low= low risk of bias/low concern of applicability, Unclear=inadequate information to make judgement							
